# Quantum chemical calculations on a unified pH scale for all phases

**DOI:** 10.1186/1758-2946-3-S1-P23

**Published:** 2011-04-19

**Authors:** SK Goll, D Himmel, I Leito, I Krossing

**Affiliations:** 1 Institute for Inorganic and Analytical Chemistry and Freiburger Materialforschungszentrum FMF, Albert-Ludwigs-University of Freiburg, 79104 Freiburg, Germany; 2 Institute of Chemistry, University of Tartu, Tartu, 50411, Estonia

## 

One for all – a unified Brønsted acidity scale…! On the basis of the absolute chemical potential of the proton a unified absolute pH scale universally applicable in the gas phase, in solution and the solid state is introduced [1]. This scale allows to directly compare acidities in different media and to give a thermodynamically meaningful definition of superacidity and can be used in all areas where proton activity changes during use, e.g. in proton-induced catalytic reactions, hydrocarbon processing, fuel cells, in the biological proton pump, and others. For calculating acidities we developed and applied several models based on quantum chemistry (modified G3, MP2/def2-QZVPP, MP2-extrapolated CCSD(T)/aug’-cc-pVDZ→QZ, COSMO@BP86/def-TZVP, etc.).

Our investigations also point out the inadequateness of the established GA scale, which in contrast to our unified acidity scale does not take into account the pressure dependent speciation in the gas phase.

**Figure 1:**
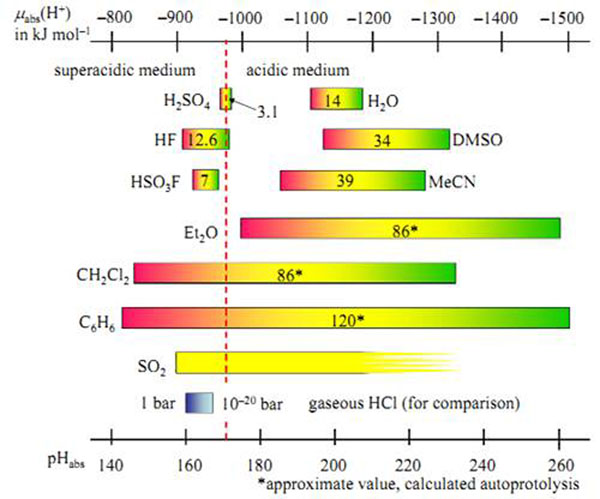
Comparison of the protochemical windows of different media.

Below the accessible absolute Brønsted acidities given by their μabs(H+) or so-called pHabs values in different media as expressed by the width of their protochemical window (= pKAP) are illustrated (Figure 1).
